# A Coarse-to-Fine Geometric Scale-Invariant Feature Transform for Large Size High Resolution Satellite Image Registration

**DOI:** 10.3390/s18051360

**Published:** 2018-04-27

**Authors:** Xueli Chang, Siliang Du, Yingying Li, Shenghui Fang

**Affiliations:** 1 School of Resource and Environmental Sciences, Wuhan University, Wuhan 430074, China; xl_whu@foxmail.com; 2 Collaborative Innovation Center Of Geospatial Technology, Wuhan University, Wuhan 430074, China; 3 School of Remote Sensing and Information Engineering, Wuhan University, Wuhan 430074, China; dusi@whu.edu.cn; 4 Institute of Remote Sensing Information of Beijing, Beijing 100000, China; ceode.ly@foxmail.com

**Keywords:** geometric SIFT, multi-thread processing, registration, large size high-resolution satellite image

## Abstract

Large size high resolution (HR) satellite image matching is a challenging task due to local distortion, repetitive structures, intensity changes and low efficiency. In this paper, a novel matching approach is proposed for the large size HR satellite image registration, which is based on coarse-to-fine strategy and geometric scale-invariant feature transform (SIFT). In the coarse matching step, a robust matching method scale restrict (SR) SIFT is implemented at low resolution level. The matching results provide geometric constraints which are then used to guide block division and geometric SIFT in the fine matching step. The block matching method can overcome the memory problem. In geometric SIFT, with area constraints, it is beneficial for validating the candidate matches and decreasing searching complexity. To further improve the matching efficiency, the proposed matching method is parallelized using OpenMP. Finally, the sensing image is rectified to the coordinate of reference image via Triangulated Irregular Network (TIN) transformation. Experiments are designed to test the performance of the proposed matching method. The experimental results show that the proposed method can decrease the matching time and increase the number of matching points while maintaining high registration accuracy.

## 1. Introduction

Image matching is a vital process that acquires the correct matched points between two images of the same scene, which may have been acquired at the same time or at different times by one or more sensors from the same viewpoint or different viewpoints [1]. Image matching has been extensively employed in many fields, including image fusion, environmental surveillance, change detection, and image orientation. In recent decades, the spatial resolution has increased significantly. A high-resolution satellite image can be several hundred megapixels in size and can occupy several spectral bands. Large size high resolution satellite (HR) images can provide detailed information [2]. However, large size HR images also bring new challenges in remote-sensing image matching. Huo reported that scale-invariant feature transform (SIFT) can extract approximately 400 MB of SIFT features from a typical 20,000 × 20,000 image. It will consume a considerable memory and time on such large number of features [2]. In HR images, ubiquitous repetitive structures (such as roads and rivers) are represented, particularly in the urban scene, which hinders feature matching and outlier removal [3]. Large size HR image matching is challenging due to low efficiency and effectiveness.

Current matching methods can be broadly classified into two categories: area-based methods and feature-based methods [4]. In area-based methods, a predefined size window is statistically compared with the same size window in the reference image. The centers of the matched windows are employed as candidate points [5]. The area-based methods are only suitable for images with minimal distortion. They cannot handle terrain areas and are sensitive to significant image distortions and intensity changes [6]. Also, the similarity measure has high computational complexity, which reduces the speed of area-based methods. Area-based methods show some difficulty in HR satellite image matching because HR satellite images contain local distortions and intensity changes because different sensors have different paths, angles, and terrain relief. Feature-based methods extract control points (CPs) based on representative points, e.g., line intersections, starting and ending points of lines, or centroid pixels of close-boundary regions [7]. The feature-based methods can be applied, even the images have some distortions and geometric differences [8]. SIFT is capable of extracting distinctive invariant features from images and can be applied to perform reliable matching across a range of affine distortion and change in illumination [9]. Due to the limitation of CPU memory, the application of SIFT to large size HR images will directly cause the system to be “out of memory”. Speed up Robust Features (SURF) is a solution that enables very fast computation of detectors using integral images and a Hessian matrix [10]. PCA-SIFT utilized principal component analysis (PCA) to decreases the dimension of feature vectors and improves the matching efficiency [11]. SURF and PCA-SIFT are superior to SIFT in efficiency, and SIFT is superior to SURF and PCA-SIFT in case of scale, rotation, and blur. These algorithms can only slightly decrease the computational cost. Fortunately, the SIFT implementation designed on a graphic processing unit (GPU) can achieve considerable computational savings [12]. The performance of SIFTGPU is nearly real-time for small and medium images. Due to the limitations of GPU memory, SIFTGPU first down-samples original images such that they can be processed within the memory capacity of the CPU or GPU [13]. However, this will cause information loss, and fewer features can be extracted from images.

The majority of SIFT-based approaches are designed for small size images, and approaches related to large size HR image registration are less reported. Gong et al. proposed a coarse-to-fine registration scheme where the coarse step is implemented by SIFT and fine step is implemented by mutual information [14]. Huo proposed a coarse-to-fine strategy and block-wise SIFT match for HR image matching [2]. The geometry is acquired using low-resolution matching. However, image information is lost at low resolution. With noise and intensity differences, the acquisition of the correct match points from the incorrect points is difficult. Sharma proposes a coarse-to-fine strategy which combine Harris detector with SIFT descriptor. Harris detector is a faster corner detection method than SIFT detection [15]. Zhang et al. applied a coarse-to-fine matching strategy for HR images registration [16]. In coarse processing, the coarse transformation between images is estimated. In fine processing, BRIEF feature is used to match more CPs. BRISK is a fast binary matching method as it can be 13 times faster than SIFT. However, it is sensitive to image scale, rotation, and intensity change. Chen proposed an automatic image registration method where the coarse step is implemented by SIFT and the fine step is implemented by NCC [17]. These methods all use a coarse-to-fine scheme, decreasing the memory usage and significantly improving efficiency but not consider the matches and the distribution. Wang developed an ASIFT-based local registration method for stereo satellite image pairs [18]. ASIFT was used for acquiring correspondences on input images. An improved random sample consensus was proposed to remove outliers robustly. Lee developed an algorithm to match satellite images based on adaptive block processing to increase the number of features and improve the distribution quality, but the time duration remained large [19]. Long used the geo-relationship between satellite images as a priori information to improve the matching performance [20]. The geometric model between images needs to be entered in advance. Han proposed a coarse-to-fine matching method for HR image matching which can extract a suitable number of evenly distributed matched points [21,22]. However, it is designed for small size HR images.

In this paper, we focus on addressing the matching difficulties caused by the overwhelming increase in image size. A geometric constraint SIFT method with a coarse-to-fine scheme is proposed to improve the matching performance and decrease the computing time. In the coarse procedure, the images are downsampled to reduce the memory burden. The SR-SIFT is used to match CPs between the downsampled images. The coarse transformation between images is then estimated. In the fine procedure, the reference image is divided into blocks, and the corresponding blocks in the sensing image are determined by the coarse transformation. In each block, a geometric SIFT is proposed to match more CPs. Finally, the sensing image is rectified to the coordinate of reference image via TIN transformation. The rest of this paper is organized as follows: Section 2 discusses the proposed HR satellite registration method, Section 3 describes the experiments and analyzes the experimental results, and Section 4 summarizes the proposed methodology and results.

## 2. Methodology

**Figure 1:**
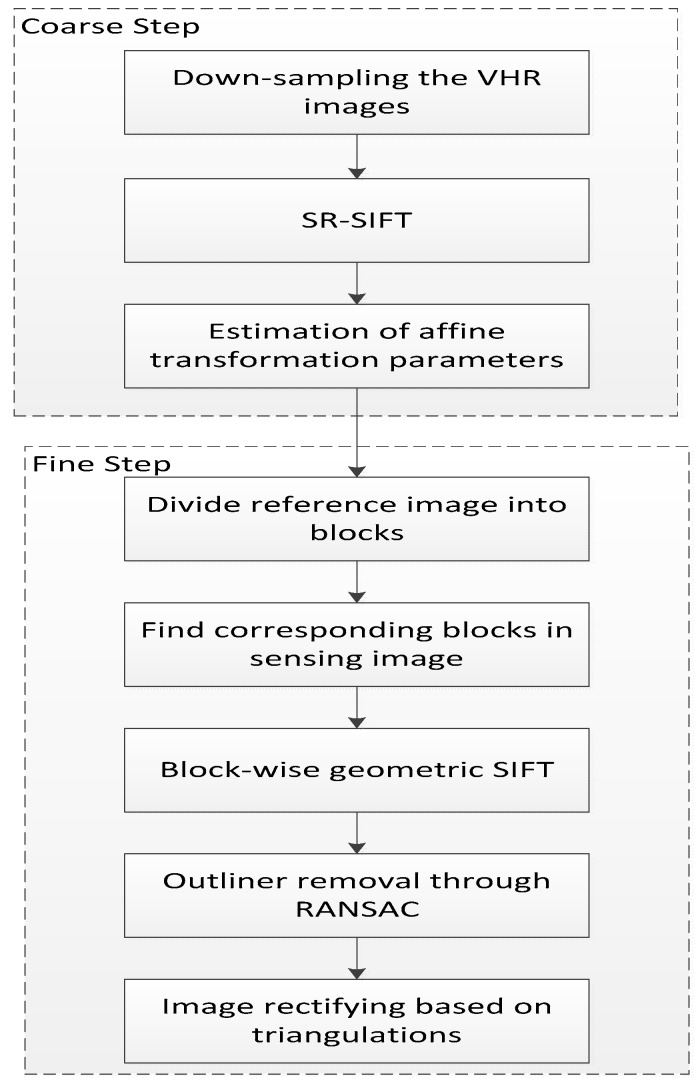
Flowchart of the proposed method.

The proposed large size HR image registration method is a coarse-to-fine matching method. In the coarse matching stage, the image is downsampled to lower resolution, and the images are matched by a robustness matching method (SR-SIFT). The coarse geometric relation between images is estimated according to the matching points. In the fine matching stage, the reference image is divided into blocks, and the corresponding blocks in the sensing image are detected by coarse geometric relation. In each block, the proposed geometric SIFT is used for matching. After all blocks are matched, the sensing image is resampled to the reference image coordinate by TIN transformation. The flow chart of the proposed method is shown in Figure 1.

### 2.1. Coarse Matching

The purpose of the coarse matching is to find the coarse geometric transformation between images. We use SIFT as it can be implemented efficiently for images of low resolution. The down-sampling level n is defined as:

(1) $$n=\log_{2}\left(N/M\right)$$

where *N* is the minimum of the width and height of the reference image and sensing image. *M* is the minimized size of the image after down-sampling. In this paper, we set the value of *M* to 800.

The image pairs are downsampled to low resolution leading to the loss of information. With information loss and noise, the acquisition of the correct match points from the incorrect points is difficult. The random sample consensus (RANSAC) is used to remove the outliers. RANSAC will fail if the correct matching is low, and the SIFT matching will fail. The SR-SIFT algorithm is a robust matching method which can raise the correct match rate in remote sensing images matching application [23].

The SR-SIFT is used to improve the correct matching rate. Given a match pair $m_{1}\left(x_{1},y_{1},\sigma_{1}\right)$, $m_{2}\left(x_{2},y_{2},\sigma_{2}\right)$, σ is the scale of the match point. The scale difference (SD) of the match pair is defined as:

(2) $$SD\left(m_{1},m_{2}\right)=\left|\sigma_{1}-\sigma_{2}\right|$$

**Figure 2:**
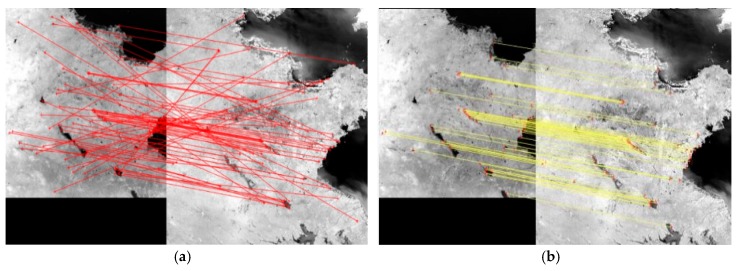
Coarse matching result. (**a**) The coarse matching result by SIFT; (**b**) The coarse matching result by SR-SIFT.

A histogram of SDs is formed. According to the initial scale difference between images, the correct matches’ *SD* can be larger than those of mismatches. The peak in the histogram is noted as PSD. (PSD−W, PSD+W) is extracted, where W is a constant value and is set between 0.20 and 0.35. If the SD of a match is located in this scope, it will be reserved. Figure 2a is the matching result by SIFT, there are many mismatching points. Figure 2b is the matching result by SR-SIFT, almost no mismatch points exist, proving the robustness of the SR-SIFT.

The matches that satisfy the scale restriction criteria are selected as tie points. Then RANSAC is used to further remove outliers and obtain CPs. Finally, the coarse transformation between images is estimated using CPs. The affine transformation model is as follows:

(3) $$\left[\begin{array}{c} x_{s}\\ y_{s}\\ 1 \end{array}\right]=\left[\begin{array}{ccc} a_{11} & a_{12} & a_{13}\\ a_{21} & a_{22} & a_{23}\\ 0 & 0 & 1 \end{array}\right]\left[\begin{array}{c} x_{r}\\ y_{r}\\ 1 \end{array}\right],$$

where $\left(x_{r},y_{r}\right)$ represents the coordinates of the pixel in the reference image, $\left(x_{s},y_{s}\right)$ represents the coordinates of the pixel in the sensing image, and $a_{11}\ldots a_{23}$ are the affine transformation parameter. The obtained affine transformation parameters are used as geometric constraint to guide matching in next fine matching.

### 2.2. Fine Matching 

#### 2.2.1. Block Division

The direct application of SIFT to these large size HR images will produce an “out of memory” message. To overcome this problem, block-based methods for large size HR, in which the entire image is divided into small-size image blocks, have been proposed [17]. A smaller image block incurs less computational complexity for convolution and sampling.

**Figure 3:**
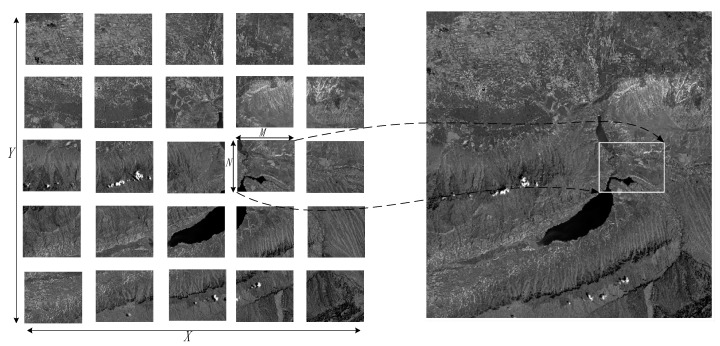
Divide the reference image into blocks and predict the corresponding block in the sensing image.

The entire reference image X∗Y is divided into smaller image blocks as shown in Figure 3. In this paper, the block size is set to 1024 × 1024. The affine transformation parameters are used as geometric constraint to find the corresponding matching block in the sensing images. For each block image, the four corners $\left(x_{i1},\;y_{i1}\right)$, $\left(x_{i2},\;y_{i2}\right)$, $\left(x_{i3},\;y_{i3}\right)$, $\left(x_{i4},\;y_{i4}\right)$ are projected onto the sensing image by affine transformation parameter with Equation (3), as shown in Figure 3. After the projection, the minimum enclosing rectangle (MER) of the four projected corners is determined. Since the affine transformation parameters are acquired at low resolution, they do not produce accurate relationship between images. The size of the MER in the sensing image should be expanded. The expanded size should not be excessive to avoid redundant feature descriptors. The expanded size is thus set to 20 pixels. The image matching is performed in each block. With block division, the memory problem can be addressed.

#### 2.2.2. Geometric SIFT

The feature-matching algorithm adopts minimum Euclidean distance on the vector for each key point in one image to find the nearest neighbor as its corresponding key point in the other image. The feature description vector of the sensing image can be expressed as $\left\{S_{i},i=1,\;2,\ldots ,m\right\}$, and the reference image feature description vector can be expressed as $\left\{R_{j},j=1,\;2,\ldots ,n\right\}$. The KNN (K-Nearest Neighbor, k = 2) algorithm is used to search the nearest and the second nearest key points in set $\left\{S_{i}\right\}$ for the key point $R_{j}$. If the Euclidean distance ratio is smaller than an empirical value (Lowe takes the value 0.8) then the key point R and the nearest key point can be regarded as a match point

There are two main methods for feature retrieval. One is the linear scan or the exhaustive search. By calculating the Euclidean distance one by one to find the nearest and second nearest key points, the exhaustive search is slow. The other method is based on constructing the data index, dividing the search space hierarchically and realizing fast matching. An example is the k-d tree, which consumes less time than the former method. However, when the dimension of the data is greater than 20, the performance of the k-d tree decreases dramatically. Lowe proposed a BBF (Best Bin First) search method, which is a modified version of the k-d tree method. However, the runtime is still large. 

**Figure 4:**
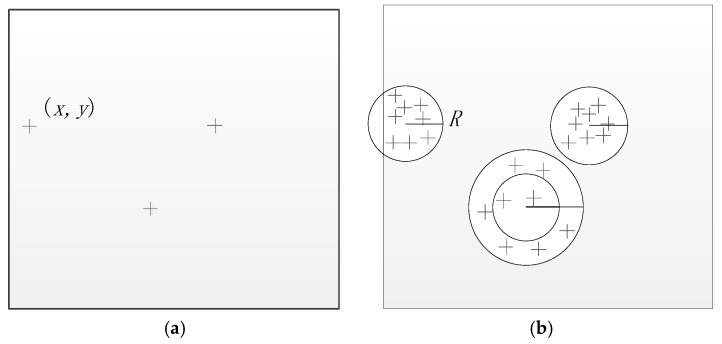
Geometric SIFT matching method which just searches the key points in the predict area. (**a**) The left image with the key point $\left(x_{r},y_{r}\right)$; (**b**) The right image with the circle of radius R in $\left(x_{s},y_{s}\right)$.

The current feature matching method searched all of the key points on the image. Here, considering the coarse registration relationship between images is obtained at coarse matching step, the paper proposes a search matching method which just searches the key points in the predict area. As shown in Figure 4, the reference image matches with the sensing image in the predict area. The key point $R_{i}$ position is located in $\left(x_{r},y_{r}\right)$ in the reference image, and the predicting matching area is located at the point of the pixel coordinate $\left(x_{s},y_{s}\right)$ within the radius R of the circle. $\left(x_{s},y_{s}\right)$ is calculated by $\left(x_{r},y_{r}\right)$ according to the coarse registration relationship using Equation (3). Matching the keypoint $R_{i}$, the search can be confined to the predicting area in the sensing image as shown in Figure 4b rather than on the global image. Because the matching search area decreases, the matching computational complexity is decreasing and running time is speeding up. The following situation may affect the search matching method. If the key point is located on the image boundary, the possible matching circle area exceeds the image boundary; however, if the number of the key points in this possible circle area is sufficient (more than 20 here), then the proposed search matching method can proceed. The adequate key points here guarantee that the KNN algorithm searches the nearest and the second nearest key point in the possible circle area. When due to a low contrary texture in one possible circle area the number of key point is inadequate, then the radius R can be expanded, ensuring sufficient key points to search. Considering the coarse relationship between images is not accuracy, and the R is set to 50 pixels. The process of the possible circle area matching method is as follow:

(1) Extract the SIFT keypoints: the sensing image keypoint set $\left\{S_{i},i=1,\;2,\ldots ,m\right\},$ and the reference image key point set $\left\{R_{j},j=1,\;2,\ldots ,n\right\}$.
(2) Obtain the matching keypoint set $\{{\mathrm{MS}}_{j}\},j=1,\;2,\ldots ,n$. Map the key point $R_{j}$ coordinate (x,y) to the sensing image, and the key point $S_{i}$ located in the radius R of the circle is put into the matching key point set $\left\{{\mathrm{MS}}_{j}\right\}$. If the key point number is less than 20, expand the circle R until key point number is more than 20.
(3) Search each the key point $R_{j}$ in the predicting matching set $\left\{{\mathrm{MS}}_{j}\right\}$.

The proposed geometric matching method reduces the key point searching area. If the key points are evenly distributed around the image, the time complexity is $O\left(N\times N\times \pi \times \frac{R^{2}}{10,240^{2}}\right)$ while the k-d tree time complexity is O(NlogN). To match in the circle of radius R, not only does the computational complexity decrease but also some wrong corresponding points would be eliminated by the geometric constraint before matching.

**Figure 5:**
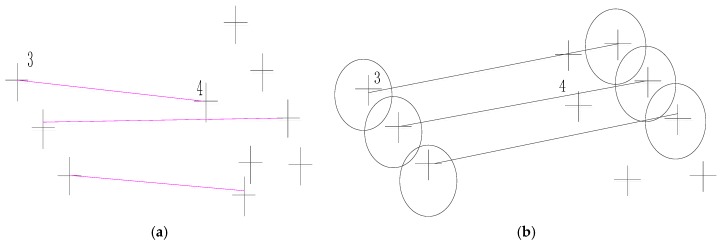
The advantage of geometric SIFT. (**a**) The error match points in the reference image; (**b**) The error match point in the registering image and the projection with a circle of radius R by the points 3 and 4 in the reference image.

Figure 5 is the error matching result between local images by SIFT. In Figure 5a, point pair ID 3 and 4 are a mismatch pairs. By the geometric matching method, the point 4 in Figure 5b is not in the predicting area projected with a circle of radius R by the point 3 in Figure 5a. The mismatch pair 3 and 4 would be eliminated previously. In addition, with the key point searching area narrowed, without interference by similar key point descriptors, the Euclidean distance ratio between the nearest neighbor and the second neighbor may be more significant, and the key point is more effective for finding the corresponding point. In other words, matching is by propagation, and by the geometrical constraint the error propagation domain is reduced from the whole image to the specified local block.

#### 2.2.3. Parallelization

Recently, computer engineering has provided the development of multi-core processors, which are composed of four or more independent cores in a single physical package [12]. OpenMP works as a set of preprocessor directives, run-time library routines, and environment variables provided to the programmer, who instructs the compiler how a section of code can be multithreaded [24]. In this paper. OpenMP has the advantage of simplicity and easy implementation. This paper employs OpenMP to multi-thread sub-size image matching applications to take advantage of multi-core CPUs.

**Figure 6:**
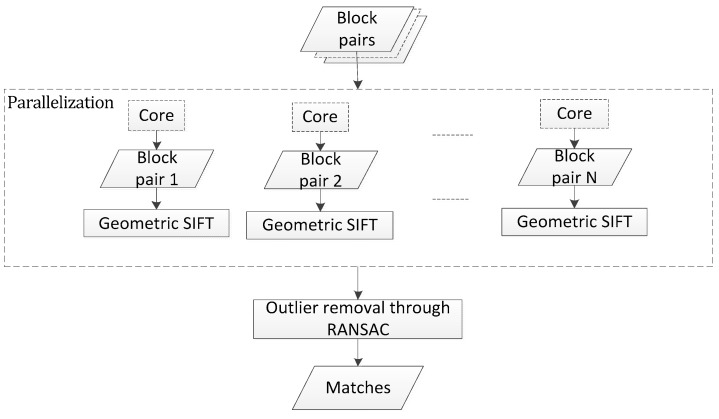
Flowchart of the parallel matching method.

In this algorithm, the matching blocks are independent and are considered to be loop-level matching. The loop-level matching can be parallelized using OpenMP. The flowchart of the multi-thread matching method is shown in Figure 6. The processor divided the reference image into blocks and obtained the corresponding blocks using affine transformation obtained in coarse matching step. Then, the processor dispersed the matching blocks to co-processors using suitable pragma directives. The parallelism is added to an application by including pragmas, such as considering the following code, which acquires the subsized block pairs that are matched with the block number N:
 
			 for (int n = 0; n < N; n++)
			       Geometric_SIFT(block, corresponding_block);
     With OpenMP, this code can be parallelized as:
		   	 #pragma omp parallel for
			     for (int n = 0; n < N; n++)
				  Geometric_SIFT (block, corresponding_block);
		  

After the block is matched, the matching points are sent to the master thread. After all blocks are matched, the master thread performs outliner removal to acquire the correct matching point.

### 2.3. Image Rectification

Local distortion and relief displacement related to landscape height occur in large size HR satellite images. Therefore, global transformation such as affine transformation, polynomial transformation and TPS transformation cannot be used to express the relationship between HR satellite images. Here, a local deformation TIN model using piecewise mapping based on transformation generated from obtained CPs. After the CPs are obtained, TINs are generated. The TIN generation is realized by the GDALTriangulationCreateDelaunay function in Geospatial Data Abstraction Library (GDAL) [25]. Each TIN of the sensing image is rectified by extracting local transformation from the three vertices of the corresponding triangle. Each pixel inside the TIN is transformed according to the estimated affine transformation parameters. The sensed image is resampled by bilinear polynomial interpolation. 

## 3. Results

**Figure 7:**
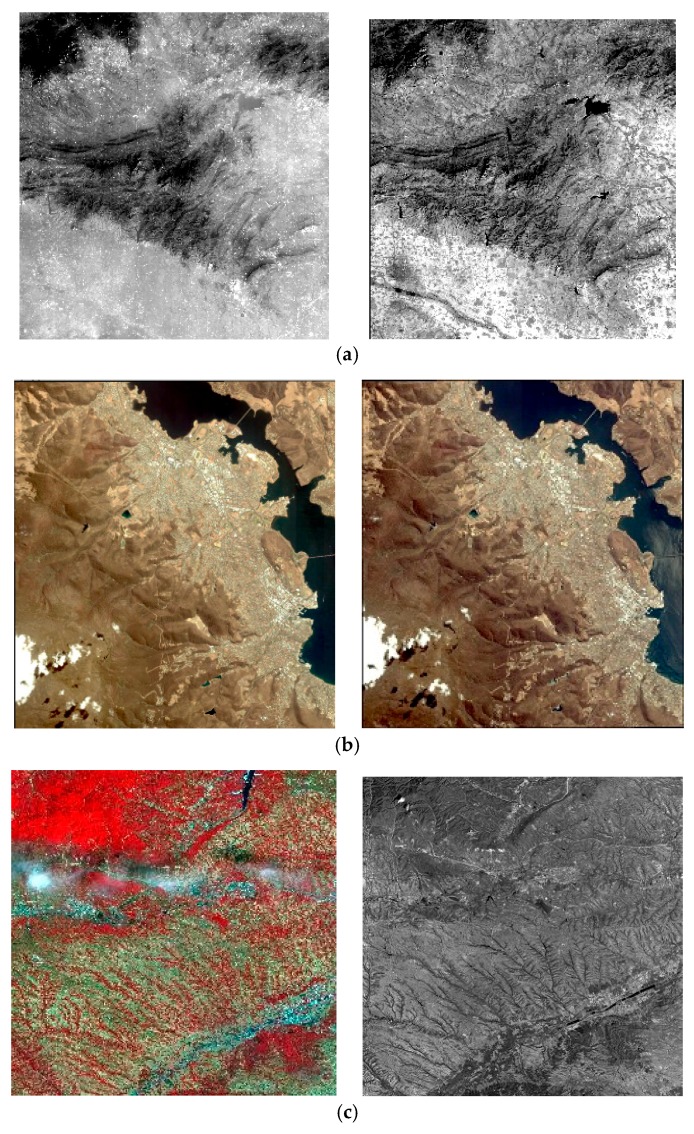
Three datasets are used for experiments which are downloaded from the homepage of ISPRS (http://www.isprs.org/data). (**a**) The dataset 1 consists of a pair of ZY3 multispectral images (5.8 m/pixel, 8817 pixel × 9215 pixel). The reference image is the green band (0.52–0.60 μm), the sensing image is near-infrared band (0.76–0.90 μm); (**b**) Dataset 2 consists of a pair of IKONOS panchromatic images (1 m/pixel, 12,122 pixel × 13,148 pixel); (**c**) Dataset 3 acquired by ZY3 consists of a multispectral (MS) image (5.8 m/pixel, 8817 pixels × 9283 pixels) and a panchromatic (PAN) image (2.1 m/pixel, 24,525 pixels × 24,410 pixels).

In this section, the proposed approach is evaluated in terms of matching performance and efficiency. Three sets of experiments are designed. The first set of experiments is to test the performance of the proposed geometric SIFT. The second experiment tests computation time to demonstrate the advantages of the multi-thread matching method using OpenMP. The third experiment compares the proposed method to other HR matching methods and shows the improvement of the proposed registration method. Three datasets which composed of multispectral and multitemporal pairs are employed for experiments, as shown in Figure 7. The proposed method is implemented using C++ and all experiments are performed on a laptop with 2.5-GHz Intel Core CPU, 8-GB memory and red hat Linux.

### 3.1. Test the Performance of The Proposed Geometric SIFT

**Figure 8:**
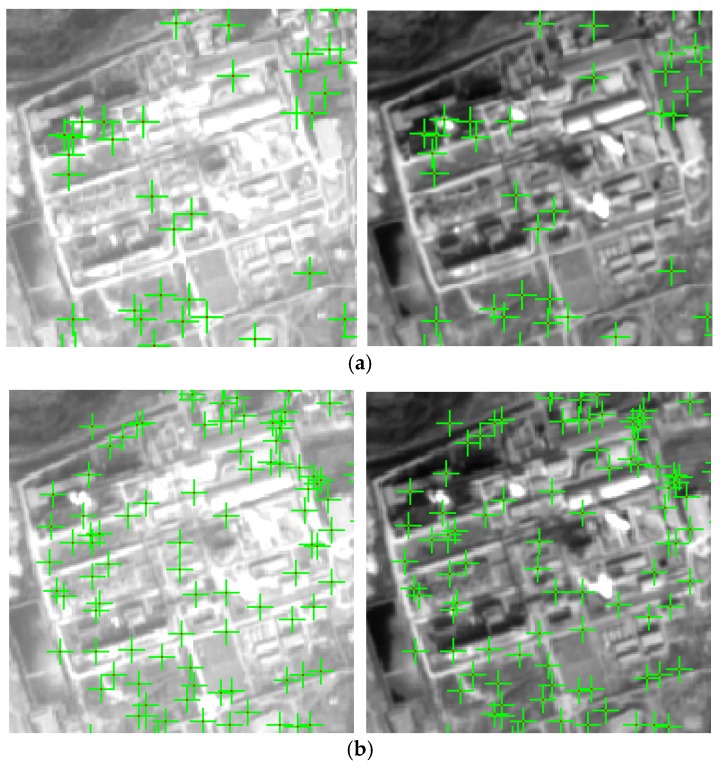
Performance of the proposed geometric SIFT. (**a**) Matching result by SIFT; (**b**) Matching result by proposed geometric SIFT.

In this section, we will test the performance of the proposed geometric SIFT and demonstrate the advantage of using geometric constraint. To reduce the computational complexity, the total image is divided into blocks, the corresponding blocks in the sensing image are determined by the affine transformation. In each block pair matching, the blocks are match by the proposed geometric SIFT. In block matching, we compare the proposed method with the SIFT. The geometric SIFT is matching the keypoints on the predicting area, the SIFT is matching the keypoints on the total block. Figure 8a is the matching result by SIFT and Figure 8b is the matching result adopting the proposed geometric SIFT. Based on visual inspection, the matching points in by the proposed geometric SIFT are more evenly distributed and greater in number, which verifies the effectiveness of the proposed geometric SIFT. 

To quantitatively evaluate the proposed method, the total number of extracted match points (TMP), the number of correct match points (CMP), the correct match rate (CMR), the number of false match points (FMP) and runtime are the measures to evaluate the proposed geometric SIFT. The definitions of CMR is as follows:

(4) $$\mathrm{CMR}=\frac{\mathrm{CMP}}{\mathrm{CMP}+\mathrm{FMP}}$$

The proposed geometric SIFT can obtain more matching points and higher CMR than SIFT (Table 1). The runtime of the proposed geometric SIFT is faster than SIFT. Geometric SIFT with area constraint is beneficial for validating the candidate matches. Some similar features that may interfere the matching ratio may be eliminated in advance. The ratio of smallest Euclidian distance to second smallest one can be more significant. The geometric SIFT can match more points. The keypoints only search in the predicting area instead of the whole block which can reduce the computation complexity, and subsequently the runtime. This experiment shows the improvement of the proposed geometric SIFT which can obtain more CPs in a shorter duration of time.

### 3.2. Efficiency

**Figure 9:**
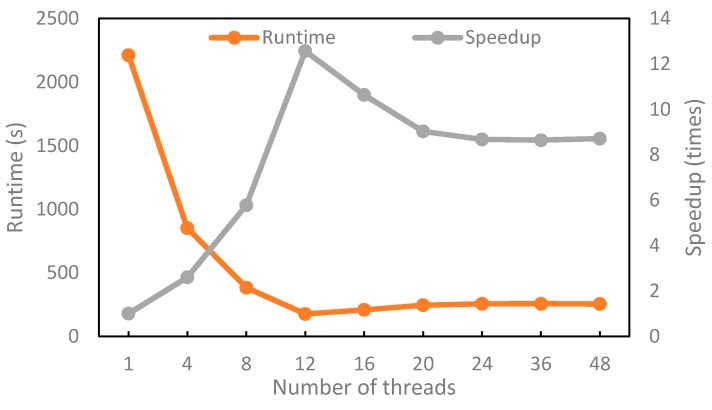
Runtime of the proposed parallelized matching method.

In this section, to demonstrate the advantages of the multi-thread matching method using OpenMP, the runtime and increase in speed were measured for the different number of cores that were used for processing. We perform the experiment using Dataset 3. Figure 9 shows the runtime and increase in speed as a function of the number of threads, *p*. 

The parallelized block-based method shows increase in speed compared with one-core processing. It achieves the highest increase in speed, 12.1 times with *p* = 12 threads. The times of increase in speed are reduced with more than *p* = 12 threads. The first factor behind these results is that the threads are locked in the data reading procedure, so the communication among threads requires additional time. The second factor is load imbalance, since block matching may have different workloads. This experiment shows that multi-thread processing can improve the running efficiency with the highest increase in speed to 12.1 times, which shows the advantage of the multi-thread matching method using OpenMP.

### 3.3. Comparison with Other HR Registration Methods

**Figure 10:**
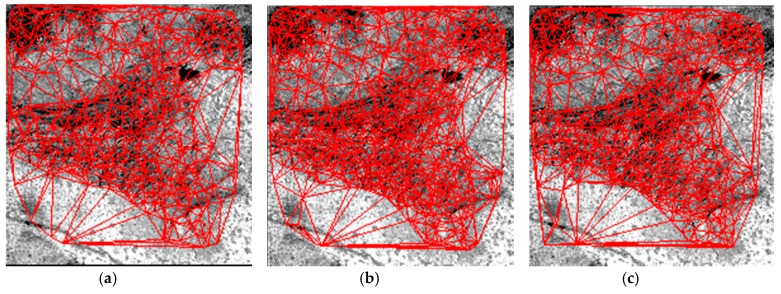
Matching result on Dataset 1. (**a**) CMPs with TIN by Lee’s method. (**b**) CMPs with TIN by Li’s method. (**c**) CMPs with TIN by the proposed method.

**Figure 11:**
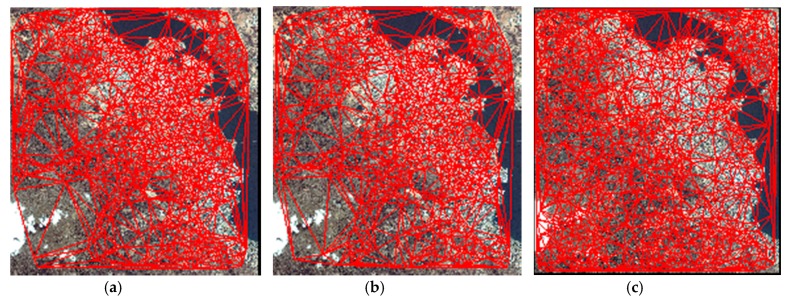
Matching result on Dataset 2. (**a**) CMPs with TIN by Lee’s method. (**b**) CMPs with TIN by Li’s method. (**c**) CMPs with TIN by the proposed method.

**Figure 12:**
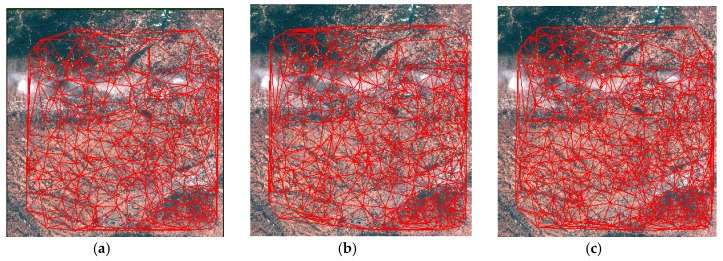
Matching result on Dataset 3. (**a**) CMPs with TIN by Lee’s method. (**b**) CMPs with TIN by Li’s method. (**c**) CMPs with TIN by the proposed method.

**Figure 13:**
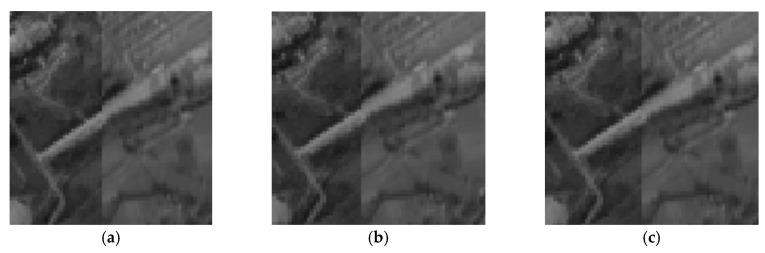
Local Registration result on Dataset 1. (**a**) Registration result by Lee’s method. (**b**) Registration result by Li’s method. (**c**) Registration result by the proposed method.

**Figure 14:**
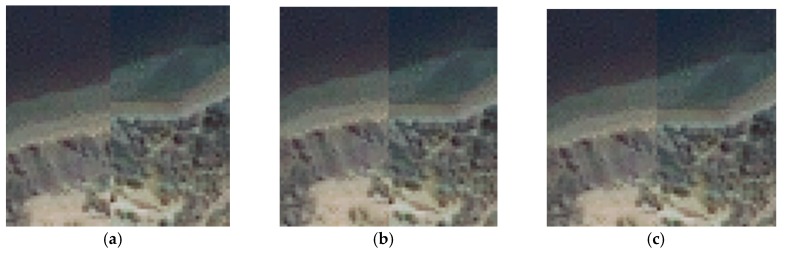
Local Registration result on Dataset 2. (**a**) Registration result by Lee’s method. (**b**) Registration result by Li’s method. (**c**) Registration result by the proposed method.

**Figure 15:**
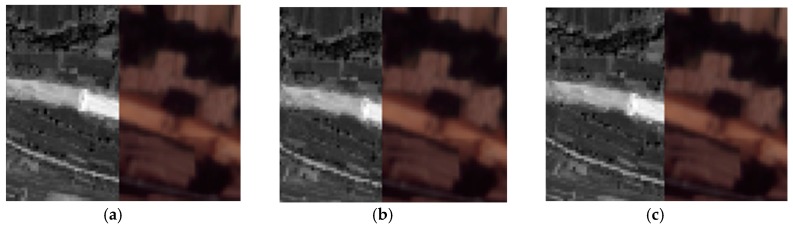
Local Registration result on Dataset 3. (**a**) Registration result by Lee’s method. (**b**) Registration result by Li’s method. (**c**) Registration result by the proposed method.

In this section, we compare the proposed HR registration method with the method proposed by Lee and the method proposed by Li. Lee uses an adaptive block processing matching strategy to matching the HR satellite images [19]. Li matches the points in the down-sampling images and the match points from low resolution image are used as interest CPs for high resolution image matching [26]. Figure 10, Figure 11 and Figure 12 are the matching result of the three HR registration method. The first column is the matching result by Lee’s method, the second column is the matching result by Li’s method and the third column is the matching result by the proposed method. The matching results are shown with TINs. To better show the results, the matching point is 20 percent of all matching points. The matching points of the proposed HR registration method are denser than the results of the methods proposed by Lee and the method proposed by Li. Figure 13, Figure 14 and Figure 15 compares the accuracy for the three HR registration method. Figure 13a, Figure 14a and Figure 15a are the local registration results by Lee’s method. Figure 13b, Figure 14b and Figure 15b are the local registration results by Li’s method. Figure 13c, Figure 14c and Figure 15c are the local registration results by proposed method. The registration results of Lee’s and Li’s methods are inaccurately aligned in contrast to the correct registration results of the proposed method. The registration accuracy of the proposed method is thus higher than that of Lee’s and Li’s methods.

To compare the algorithms quantitatively, the CMP, CMR, runtime, registration accuracy are compared. The RMSE is used to measure the registration accuracy and is defined as:

(5) $$\mathrm{RMSE}=\sqrt{\frac{1}{n}\sum_{i=1}^{n}{\left(p_{i}^{\prime }-p_{i}\right)}^{2}}$$

where $p_{i}^{\prime }$ and $p_{i}$ are match points between reference image and rectified image automatically extracted by ENVI software and *n* represents the number of match points. If the reference image and sensing image are registered accurately, point $p_{i}^{\prime }$ in the reference image and point $p_{i}$ in the rectified image are in the same position.

From Table 2, Table 3 and Table 4, we can see that the CMP and CMR values are greater than that of Lee’ method and Li’s method. The proposed method is a coarse-to-fine matching method, and the result of coarse matching is used as geometric result for geometric SIFT fine matching. With geometric constraint, the key point searching area is narrowed. Without interference by similar key point descriptors, the Euclidean distance ratio between the nearest neighbor and the second neighbor may be more significant, and the key point is more effective for finding the corresponding point. The registration accuracy of the proposed HR matching method is better than that of Lee’s method and Li’ method. Not only is the CMP number of the proposed method greater than that of Lee’s and Li’s methods, but the CMPs are also well distributed in the proposed method. The greater number of TIN and better distributed TIN between images, the higher registration accuracy will be. The registration accuracy of the proposed method is thus higher than that of Lee’s method and Li’s method. Additionally, the proposed HR matching has the shortest time runtime. The proposed method just search the key point in the predicting area instead of the whole block. As search computation complexity decreased, the runtime decreased. These results show the advantages of the proposed HR registration method.

## 4. Conclusions

An effective and efficient matching method is proposed in this paper for the registration of HR images. The coarse-to-fine matching strategy, the block matching method, geometric SIFT, multi-threads processing and TIN transformation are proposed in the HR image registration method. The block matching method and geometric SIFT benefits from the coarse registration at low resolution images. The block matching method can overcome the memory problem. The geometric SIFT match the keypoints in the prediction area. With area constraint, the search complexity is decreased and the false matches may be eliminated before matching. The efficiency is improved, and our parallelized approach can achieve the highest increase in speed to 12.1 times. The TIN transformation is a local transformation which is used for HR images registration. Experimental results show that the proposed method can match more CPs at a shorter runtime while maintaining a high registration accuracy.

## Figures and Tables

**Table 1:** Test the performance of geometric SIFT.

	TMP	CMP	CMR	Runtime(s)
SIFT	451	282	62.5%	6.2
Geometric SIFT	610	510	85%	4.1

**Table 2:** Comparison of the three HR registration method on dataset 1.

	CMP	CMR	RMSE (Pixel)	Runtime(s)
Lee’s method	3431	73.1%	0.51	1006
Li’s method	4621	79.2%	0.42	931
Proposed method	6123	85.1%	0.31	72

**Table 3:** Comparison of the three HR registration method on dataset 2.

	CMP	CMR	RMSE (Pixel)	Runtime(s)
Lee’s method	5612	75.1%	1.27	1461
Li’s method	8821	82.3%	0.87	1131
Proposed method	10,123	87.2%	0.69	84

**Table 4:** Comparison of the three HR registration method on dataset 3.

	CMP	CMR	RMSE (Pixel)	Runtime(s)
Lee’s method	4209	70.4%	0.96	1607
Li’s method	5322	76.5%	0.74	1408
Proposed method	7533	79.4%	0.52	102
